# Functional and Neurochemical Identification of Ghrelin Receptor (GHSR)-Expressing Cells of the Lateral Parabrachial Nucleus in Mice

**DOI:** 10.3389/fnins.2021.633018

**Published:** 2021-02-15

**Authors:** Marie V. Le May, Fiona Peris-Sampedro, Iris Stoltenborg, Erik Schéle, Tina Bake, Roger A. H. Adan, Suzanne L. Dickson

**Affiliations:** ^1^Department of Physiology/Endocrinology, Institute of Neuroscience and Physiology, The Sahlgrenska Academy at the University of Gothenburg, Gothenburg, Sweden; ^2^Department of Translational Neuroscience, UMC Utrecht Brain Center, Utrecht University, Utrecht, Netherlands

**Keywords:** GHSR, ghrelin, parabrachial nucleus, body weight, food intake and food choice, glutamate, PACAP, CGRP

## Abstract

The lateral parabrachial nucleus (lPBN), located in the pons, is a well-recognized anorexigenic center harboring, amongst others, the calcitonin gene-related peptide (CGRP)-expressing neurons that play a key role. The receptor for the orexigenic hormone ghrelin (the growth hormone secretagogue receptor, GHSR) is also abundantly expressed in the lPBN and ghrelin delivery to this site has recently been shown to increase food intake and alter food choice. Here we sought to explore whether GHSR-expressing cells in the lPBN (GHSR^*lPBN*^ cells) contribute to feeding control, food choice and body weight gain in mice offered an obesogenic diet, involving studies in which GHSR^*lPBN*^ cells were silenced. We also explored the neurochemical identity of GHSR^*lPBN*^ cells. To silence GHSR^*lPBN*^ cells, *Ghsr-IRES-Cre* male mice were bilaterally injected intra-lPBN with a Cre-dependent viral vector expressing tetanus toxin-light chain. Unlike control wild-type littermates that significantly increased in body weight on the obesogenic diet (i.e., high-fat high-sugar free choice diet comprising chow, lard and 9% sucrose solution), the heterozygous mice with silenced GHSR^*lPBN*^ cells were resistant to diet-induced weight gain with significantly lower food intake and fat weight. The lean phenotype appeared to result from a decreased food intake compared to controls and caloric efficiency was unaltered. Additionally, silencing the GHSR^*lPBN*^ cells altered food choice, significantly reducing palatable food consumption. RNAscope and immunohistochemical studies of the lPBN revealed considerable co-expression of GHSR with glutamate and pituitary adenylate cyclase-activating peptide (PACAP), and much less with neurotensin, substance P and CGRP. Thus, the GHSR^*lPBN*^ cells are important for diet-induced weight gain and adiposity, as well as in the regulation of food intake and food choice. Most GHSR^*lPBN*^ cells were found to be glutamatergic and the majority (76%) do not belong to the well-characterized anorexigenic CGRP cell population.

## Introduction

The parabrachial nucleus (PBN), located in the pons, is known to relay a broad range of sensory information to forebrain regions (Saper and Loewy, 1980; Fulwiler and Saper, 1984; Bernard et al., 1993; Krout and Loewy, 2000), including information about taste (Norgren and Leonard, 1971; Geran and Travers, 2009; Tokita and Boughter, 2016), as well as aversive information such as visceral malaise (Bernard et al., 1994; Yamamoto et al., 1995; Thiele et al., 1996; Sakai and Yamamoto, 1997) and pain (Gauriau and Bernard, 2002). Part of this neurocircuitry includes an anorexigenic cell group in the lateral parabrachial nucleus (lPBN), that receives GABAergic signaling from the arcuate nucleus (ARC) agouti-related peptide (AgRP)/neuropeptide Y (NPY)/gamma-aminobutyric acid (GABA) neurons, a pathway crucial for feeding to occur (Wu et al., 2009). Diphtheria toxin-mediated ablation of AgRP neurons induced starvation and feeding was restored by chronic stimulation of GABAergic signaling at the level of the lPBN. Calcitonin gene-related peptide (CGRP) neurons in the lPBN that project to the central nucleus of the amygdala (CeA) were then identified as the profoundly anorexigenic pathway inhibited by AgRP/NPY/GABA neurons to drive feeding, as revealed by chemogenetic and optogenetic studies in Calca-Cre mice injected with Cre-dependent viral vectors into the lPBN (Carter et al., 2013). The lPBN CGRP cells were subsequently found to mediate conditioned taste aversion (Carter et al., 2015) and to control meal termination (Campos et al., 2016). These findings highlight the key role of lPBN CGRP neurons in the regulation of feeding behaviors.

There are indications that the lPBN forms part of the neurocircuitry engaged by (circulating) appetite-regulating hormones. Receptors for anorexigenic peptides such as leptin and glucagon-like peptide 1 (GLP-1) are expressed in the lPBN (Merchenthaler et al., 1999; Scott et al., 2009) and local microinjection of these peptides into this area in rats has been shown to reduce food intake (Alhadeff et al., 2014a,b; Richard et al., 2014). Peptides of the YY family (PYY) have been shown to signal here (likely *via* Y1 receptors) to increase food intake (Alhadeff et al., 2015).

Interestingly, the receptor for the appetite-stimulating hormone ghrelin (the growth hormone secretagogue receptor, GHSR) is abundantly expressed in the lPBN, revealed by *in situ* hybridization studies (Zigman et al., 2006). More recently, this distribution was faithfully reproduced in studies utilizing a novel Cre-driver line of mice (*Ghsr-IRES-Cre* mice) crossed onto two different reporter strains (Mani et al., 2017). The GHSR-expressing cells were located in more dorsal parts of the lPBN, which corresponds to the location of glutamate (Niu et al., 2010) as well as other peptides such as pituitary adenylate cyclase-activating peptide (PACAP), neurotensin and substance P as described in the Allen Brain Atlas^1^ (Ng et al., 2009), but less so for CGRP which is distributed more ventro-laterally in this nucleus (Carter et al., 2013).

Ghrelin is a stomach-derived hormone known to engage the orexigenic AgRP/NPY neurons of the ARC and the dopaminergic cells of the ventral tegmental area, two key hubs in the feeding circuitry (Skibicka and Dickson, 2011; Perello and Dickson, 2015). Ghrelin has been shown to increase food intake (Wren et al., 2000) and alter food choice (Schéle et al., 2016). Recently, we provided evidence that ghrelin signaling in the lPBN may contribute to these effects since lPBN injection of ghrelin increased food intake and altered food choice in rats (Bake et al., 2020). Orexigenic and pro-obesity effects of intra-lPBN delivery of ghrelin have since been reported in mice and shown to require endogenous GHSR signaling, since they were absent in GHSR knockout mice (Zhang et al., 2020).

In the present study, we first sought to explore the impact of loss of GHSR signaling selectively in the lPBN on body weight progression, food intake and food choice. Based on ghrelin injection studies (Bake et al., 2020; Zhang et al., 2020), silencing GHSR^*lPBN*^ cells could be expected to have the opposite effect to ghrelin injection (i.e., reduce food intake and body weight gain). There is, however, considerable overlap and redundancy in systems ensuring sufficient food intake to sustain energy balance and it is therefore unclear whether selective silencing of GHSR^*lPBN*^ cells would be sufficient to reduce food intake and limit body weight gain. Arguably, food choice may be less critical than food intake for survival, at least in the short term, and there may be less need for overlap and redundancy in the pathways regulating it. Therefore, our main hypothesis was that GHSR^*lPBN*^ cells may play an important role in food choice. To silence GHSR^*lPBN*^ cells, we used *Ghsr-IRES-Cre* mice (Mani et al., 2017) which we injected intra-lPBN with a Cre-dependent viral vector expressing the tetanus toxin-light chain (AAV1-CBA-DIO-eGFP-Tetox-WPRE-pA) (Carter et al., 2015) to block synaptic transmission (Yamamoto et al., 2003) of the GHSR^*lPBN*^ cells and hence, silence their communication and function. We also explored the neurochemical identity of the GHSR^*lPBN*^ cells (using RNAscope and immunohistochemistry) and hypothesized that they likely include one or more of the aforementioned lPBN cell groups.

## Materials and Methods

### Animals

All studies were carried out on adult male *Ghsr-IRES-Cre* heterozygous mice crossed (or not) with ROSA26-ZsGreen reporter mice B6.Cg-Gt(ROSA)26Sortm6(CAG-ZsGreen1)Hze/J (The Jackson Laboratory; stock number 007906) as previously described (Mani et al., 2017). The original heterozygous colony was produced by Prof Jeffrey M. Zigman (UT Southwestern Medical Center, Dallas, United States) and the breeding pairs were kindly provided by Prof Zane B. Andrews (Monash University, Melbourne, Australia). The mice used in this study were bred in-house at Gothenburg University (Sweden).

Mice were housed in a 12-h light/dark cycle (lights on a 7:00) at 20 ± 2°C and 50% humidity and had *ad libitum* access to standard maintenance chow (Teklad diet 2016; Envigo, Cambridgeshire, United Kingdom) and water unless otherwise stated. All procedures were approved by the local Ethics Committee for Animal Care and Use at the University of Gothenburg (permit number: 27-2015 and 132-2016) and conducted in accordance with legal requirements of the European Community (Decree 86/609/EEC).

### Experimental Design

Thirteen heterozygous *Ghsr-IRES-Cre* (*Ghsr-Cre* Het) male mice and 8 wild-type male littermates (WT) were injected bilaterally into the lPBN with a viral vector (AAV1-CBA-DIO-eGFP-Tetox-WPRE-pA) (Carter et al., 2015). The expression of tetanus toxin light chain (Tetox), which stops synaptic transmission (Carter et al., 2015), is Cre-dependent and will only be expressed therefore in the *Ghsr-Cre* Het mice and not in the control WT mice. As a result, this strategy aims to prevent signaling of GHSR-expressing cells in the lPBN in the *Ghsr-Cre* Het mice (that we now refer to as the GHSR-silenced group) but not in WT controls.

At 1 week after surgery, the individually housed mice were introduced to a high-fat, high-sugar (HFHS) free choice diet consisting of standard chow pellets, lard (saturated animal fat; Dragsbæk, Thisted, Denmark), 9% sucrose solution (Peris-Sampedro et al., 2019) and water, as described previously (Schéle et al., 2016). Body weight and intake from each dietary component were measured daily, at the same time of the day (approximately at 12:00) for 23 days. Caloric efficiency was calculated on Day 23 of exposure to the HFHS free choice diet as follows: caloric efficiency = [body weight gain (g) / food intake (kcal)] × 100 (Rabasa et al., 2019). On days 24, 25, and 26 after viral injections, the mice were subjected to saccharin preference tests (see related section below) to explore the effect of silencing GHSR^*lPBN*^ cells on sweet taste sensitivity.

At the end of the study, mice were perfused to verify the viral vector injection sites. Bilateral epididymal fat pads were collected and weighed prior to transcardial perfusion when mice were anesthetized.

Two GHSR-silenced mice were excluded because post-mortem evaluation revealed that the injection site was off-target. Three GHSR-silenced mice were sacrificed 3 weeks post-surgery to monitor the viral vector expression and their data were included in the analysis of body weight. Another GHSR-silenced mouse showed a very strong body weight phenotype in response to Tetox and began to look weak and unhealthy 5 days before the end of the experiment. This mouse was therefore sacrificed at that time point and included in the analysis of body weight, food choice and saccharin preference. In addition, one control mouse died just after surgery. Thus, the final analysis included: (i) 8 GHSR-silenced mice and 7 control mice for the study of caloric intake, food choice and saccharin preference, (ii) 7–11 GHSR-silenced mice (11 for the first 2 weeks, then 8 and finally 7 for the 5 last days) and 7 control mice for the study of body weight development and caloric efficiency on the HFHS diet, and (iii) 7 GHSR-silenced mice and 7 control mice for the fat pad analysis.

In an additional cohort of mice (6 GHSR-silenced and 9 controls) we performed a parallel study, measuring food intake and body weight progression in mice fed regular chow (Supplementary Material 1).

In addition, four *Ghsr-IRES-Cre* wild-type male mice and five *Ghsr-IRES-Cre* heterozygous male mice on the ZsGreen reporter background (i.e., in which GHSR-positive cells express ZsGreen) were perfused to determine the neurochemical identity of the GHSR^*lPBN*^ cells using fluorescent *in situ* hybridization (RNAscope) and immunohistochemistry, respectively (see sections below).

### Stereotaxic Surgeries

All mice (26–41 g body weight) received a subcutaneous (s.c.) injection of the analgesic Rimadyl^®^ (5 mg/kg; Orion Pharma Animal Health, Sollentuna, Sweden) and were deeply anesthetized by intraperitoneal (i.p.) injection of a combination of Sedastart vet^®^. (1 mg/kg; Produlab Pharma B.V., Raamsdonksveer, The Netherlands) and Ketalar^®^ (75 mg/kg; Pfizer AB, New York City, United States) and placed in a stereotaxic frame. After exposure of the skull and application of a local anesthetic (Xylocaine 10%, AstraZeneca, Cambridge, United Kingdom), two holes were drilled and the viral vector expressing Tetox was injected (0.4 μl, 1.8 × 10^12^ particles/mL, 0.1 μl/min) into each side using a 31 gage stainless steel needle (Coopers Needle Works Ltd., Birmingham, United Kingdom) connected *via* vinyl tubing to a Hamilton syringe placed in an infusion pump. The injection volume was optimized prior to the study in order to minimize spreading of the viral vector outside the lPBN. For injection of Tetox-expressing viral vector in the lPBN, the following coordinates were used: 5.34 mm posterior to bregma; 1.3 mm lateral to the midline; 3.7 mm ventral of the skull surface at bregma. After injection, the injection needle was kept in place for an additional 7 min and then slowly retracted to ensure full diffusion from the needle tip. After surgery, mice were injected with the sedation-reversing Sedastop vet^®^. (2.5 mg/kg s.c.; Produlab Pharma B.V., Raamsdonksveer, The Netherlands), individually housed and allowed to recover for at least a week. Correct placement of the needle tip from the viral injections was confirmed post-mortem in all mice.

### Saccharin Preference Test

The mice had *ad libitum* access to a saccharin solution (0.1% w/v; Sigma-Aldrich, St. Louis, MO, United States) in addition to water for 3 h/day on two consecutive days (the first day can be considered a habituation day) (Rabasa et al., 2016) and 1 h/day on the third day. The aim of having only 1 h exposure instead of 3 h on the third day was to increase the sensitivity of the test, as explained previously (Rabasa et al., 2016). The saccharin and water bottles were checked for leakage before the test and side-switched each day to control for potential side preference. Saccharin and water bottles were weighed before and after each access session. Preference for the saccharin solution over water was calculated as follows: [ml of saccharin / (ml of saccharin + ml of water)] × 100.

### Tissue Processing

The mice were deeply anaesthetized with the mixture of Sedastart vet^®^. and Ketalar^®^ mentioned above and perfused transcardially with heparinized 0.9% saline followed by 4% paraformaldehyde (PFA) in 0.1 M PB. The brains were dissected, post-fixed overnight at 4 °C in 4% PFA solution (brains used for immunohistochemistry were post-fixed in 4% PFA containing 15% sucrose, those for RNAscope in simple 4% PFA for maximum 20 h) and cryoprotected in 0.1 M PB containing 30% sucrose (25% sucrose for RNAscope) at 4°C until cryosection. Coronal sections containing the lPBN (30 μm-thick for immunohistochemistry or 14 μm-thick for RNAscope) were then cut using a cryostat and stored in tissue storage solution (25% glycerin, 25% ethylene glycol, 50% 0.1 M PB, autoclaved for RNAscope) at −20°C until further processing.

### Neurochemical Identification of GHSR^*lPBN*^ Cells

Glutamate, pituitary adenylate cyclase-activating peptide (PACAP), substance P and neurotensin emerged as appropriate candidates as a result of literature search (Palmiter, 2018), and comparison of the location in the lPBN of the cells expressing the different neurotransmitters with that of the GHSR cells in the Allen Brain Atlas^2^ (Ng et al., 2009).

Fluorescent *in situ* hybridization using RNAscope^®^ was performed to study the potential co-expression of GHSR with glutamate (*Slc17a6* probed), PACAP (*Adcyap1* probed), substance P (*Tac1* probed) and neurotensin in the lPBN of *Ghsr-IRES-Cre* wild-type male mice (*n* = 4). All reagents were purchased from Advanced Cell Diagnostics (ACD, Hayward, CA, United States) if not stated otherwise.

The *Ghsr* probe (Cat. No. 426141-C3) contained 20 oligo pairs and targeted region 438–1385 (Acc. No. NM_177330.4) of the *Ghsr* transcript. The *Slc17a6* (glutamate transporter) probe (Cat. No. 319171-C2) contained 20 oligo pairs and targeted region 1,986–2,998 (Acc. No. NM_080853.3) of the *Slc17a6* transcript. The *Adcyap1* (PACAP-coding gene) probe (Cat. No. 405911) contained 20 oligo pairs and targeted region 676–1,859 (Acc. No. NM_009625.2) of the *Adcyap1* transcript. The *Tac1* (substance P-coding gene) probe (Cat. No. 410351-C2) contained 15 oligo pairs and targeted region 20–1,034 (Acc. No. NM_009311.2) of the *Tac1* transcript. The *neurotensin* probe (Cat. No. 420441) contained 20 oligo pairs and targeted region 2–1,188 (Acc. No. NM_024435.2) of the *neurotensin* transcript. Three-plex negative and three-plex positive control probes recognizing bacterial dihydrodipicolinate reductase, DapB (Cat. No. 320871) and PolR2A, cyclophilin and Ubiquitin (Cat. No. 320881), respectively, were processed in parallel with the target probes to ensure tissue RNA integrity and optimal assay performance.

All incubation steps were performed at 40°C using the ACD HybEz II hybridization system (Cat. No. 321462). On the day before the assay, every 6th section throughout the lPBN was mounted on SuperFrost Plus slides (631–9,483; VWR, Radnor, PA, United States), dried at room temperature, briefly rinsed in autoclaved Milli-Q purified water, air-dried and baked at 60°C overnight. From one of the animals, sections from the same region of the brain were also mounted for use with the positive and negative control probes. On the day of the assay, slides were first incubated for 7 min in hydrogen peroxide (Cat. No. 322335), submerged in Target Retrieval (Cat. No. 322001) at a temperature of 98.5–99.5°C for 7 min, followed by two brief rinses in autoclaved Milli-Q purified water. The slides were quickly dehydrated in 100% ethanol and allowed to air-dry for 5 min. A hydrophobic barrier was then created around the sections using an ImmEdge hydrophobic barrier pen (Cat. No. 310018) and the sections were incubated with Protease Plus (Cat. No. 322331) for 30 min. The subsequent steps, i.e., hybridization of the probes and the amplification and detection steps, were performed according to the manufacturer’s protocol for the tyramide-based RNAscope^®^ Multiplex Fluorescent v2 Assay (Cat. No. 323100). The *Adcyap1* and *neurotensin* probes were labeled with Opal 520 (1:500; FP1487A; PerkinElmer, Waltham, MA, United States), the *Ghsr* probe with Opal 570 (1:2000; FP1488A, PerkinElmer), and the *Slc17a6* and *Tac1* probes with Cy5 (1:2000; Akoya Biosciences, Menlo Park, CA, United States).

Sections were counterstained with DAPI and coverslipped with Prolong Diamond Antifade mounting medium (P36970; Thermo Fisher, Waltham, MA, United States) and stored in the dark at 4 °C until image acquisition.

To determine whether the GHSR^*lPBN*^ cells overlap with the CGRP-expressing population, immunohistochemistry was performed on every 6th lPBN-containing sections from *Ghsr-IRES-Cre* heterozygous male mice on the ZsGreen reporter background (*n* = 5). The sections were incubated with goat anti-CGRP antibody (1:40; Ab36001; Abcam, Cambridge, United Kingdom) overnight at 4 °C followed by Alexa Fluor 594 chicken anti-goat secondary antibody (1:200; A21468; Invitrogen, Carlsbad, CA, United States) for 1 hr at room temperature before being mounted onto glass slides and coverslipped with ProLong Diamond Antifade mounting medium. The slides were stored at 4 °C until image acquisition.

### Imaging and Image Analysis

Images of the immunohistochemical staining for CGRP and the RNAscope for GHSR, glutamate, PACAP, substance P and neurotensin were acquired using a laser scanning confocal microscope (LSM 700 inverted, Zeiss, Oberkochen, Germany) at the Centre for Cellular Imaging at Gothenburg University. A Plan-Apochromat 20x/0.8 (WD = 0.55 mm) objective was used with 3 × 2 tiling settings to image the immunohistochemical staining, while a Plan-Apochromat 40x/1.3 Oil DIC objective was used with 6 × 4 tiling and z-stack settings to image the RNAscope. We used the same image acquisition settings for all images in each study and ensured that these settings did not detect any signal in the negative control sections (sections incubated with the secondary antibody but not with the primary for immunohistochemistry and sections incubated with the negative control probe for RNAscope). The z-stack images were processed using the maximum intensity projection function in the Zen Black software (Zeiss). The final images were then stitched and the cells counted in ImageJ/Fiji (NIH, Bethesda, MD, United States). The cell counter plug-in was used to count positive cells and co-localization in the lPBN. DAPI was used to make sure any positive signal counted was indeed a cell, although we did not simultaneously check for neuronal/glial markers. Two and 4–5 lPBN sections from bregma −5.07 to −5.33 mm were used for the quantification of the immunohistochemical and the RNAscope images, respectively.

### Statistical Analysis

Data were analyzed using IBM SPSS Statistics 25 (IBM Corp., Armonk, NY, United States). All data were tested for normal distribution using a Shapiro-Wilk test and for homogeneity of variances using a Levene’s test. Throughout the 23 days of HFHS free choice diet exposure, % body weight change and total energy intake were analyzed by one-way repeated measures ANOVA (group) with the days as the within-subject factor. Bonferroni *post hoc* test was used to adjust for multiple comparisons. The body weights at the start of exposure to the HFHS diet and after 23 days for each group separately were compared using a paired sample Student’s *t*-test. Total caloric intake and food choice data on HFHS free choice diet were analyzed using a one-way ANOVA (group). A Student’s *t*-test was used to examine data from the saccharin preference test as well as the fat pads weights. The results are reported as mean ± SEM and statistical significance was set at *p* < 0.05.

## Results

### Silencing GHSR^*lPBN*^ Cells Protects Against HFHS Diet-Induced Weight Gain in Mice

**FIGURE 1:**
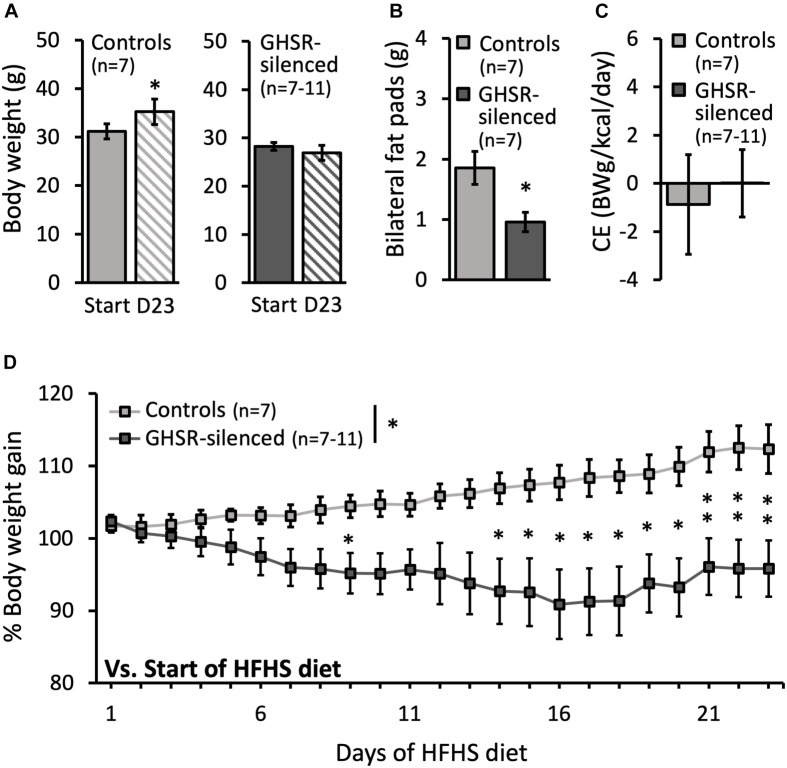
Effect of GHSR^*lPBN*^ cells Tetox-silencing on body weight, epididymal fat pads and caloric efficiency. **(A)** Body weight at the start and on day 23 (D23) of access to high-fat, high-sugar (HFHS) free choice diet for both the control group (lighter grey) and the GHSR-silenced group (darker grey). **(B)** Weight of bilateral epididymal fat pads at the end of the experiment for both groups. **(C)** Caloric efficiency of both groups on day 23 of HFHS free choice diet. **(D)** Evolution of % body weight gain of both groups on HFHS free choice diet over time (body weight at start of HFHS diet = 100%). Data shown as mean ± SEM. ^∗^*p* < 0.05 and ^∗∗^*p* ≤ 0.01.

Control mice increased their body weight over the period of HFHS exposure [HFHS Start: 31.2 ± 1.5 g and Day 23: 35.2 ± 2.6 g; *t*(6) = −3.20, *p* = 0.019] (Figure 1A). In contrast, the body weight of the GHSR-silenced mice did not increase during this period and, if anything, tended to decrease (HFHS Start: 28.2 ± 0.8 g and Day 23: 26.9 ± 1.5 g; not significant) (Figure 1A). The percentage body weight gain (with individual body weight at start of HFHS diet = 100%) was significantly reduced in the GHSR-silenced mice compared to the control mice [overall group effect on percentage body weight: *F*(1, 12) = 6.06, *p* = 0.030] (Figure 1D). Specifically, the GHSR-silenced mice had a percentage body weight significantly lower than controls on day 9 (*p* = 0.043) and on days 14–23 (Day 14 *p* = 0.045; Day 15 *p* = 0.044; Day 16 *p* = 0.026; Day 17 *p* = 0.018; Day 18 *p* = 0.019; Day 19 *p* = 0.014; Day 20 *p* = 0.008; Day 21 *p* = 0.009; Day 22 *p* = 0.007; Day 23 *p* = 0.010) (Figure 1D).

In accordance with the reduction in body weight seen in the GHSR-silenced group, these mice also had a significantly lower amount of dissected epididymal fat (0.9 ± 0.2 g) compared to controls (1.7 ± 0.3 g) [*F*(1, 12) = 5.91, *p* = 0.032] (Figure 1B).

The effects of silencing GHSR^*lPBN*^ cells to suppress food intake and decrease body weight gain could only be observed in mice fed a HFHS diet (Figure 1) and not in mice fed regular chow (Supplementary Figure 1).

### Caloric Intake Is Reduced and Caloric Efficiency Unchanged by Silencing GHSR^*lPBN*^ Cells

**FIGURE 2:**
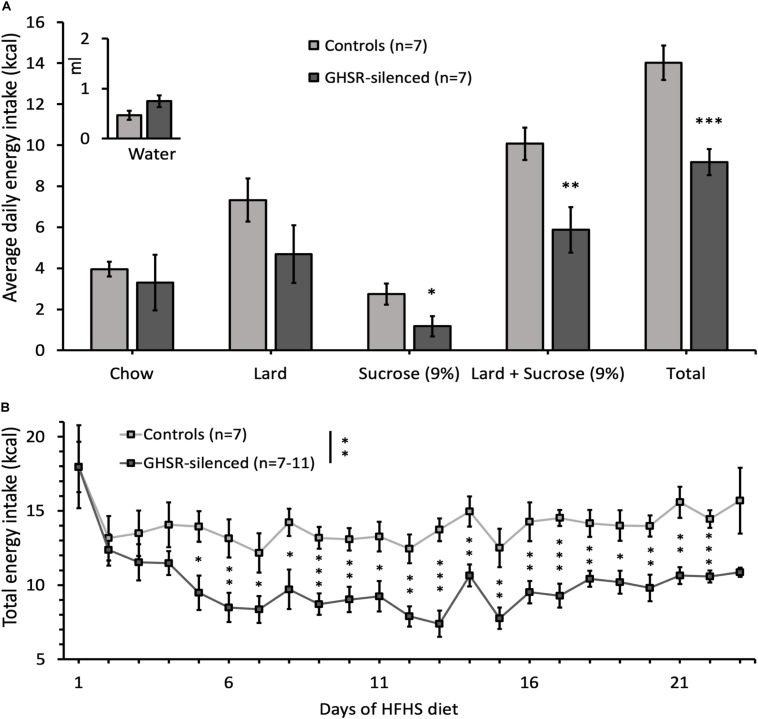
Effect of GHSR^*lPBN*^ cells Tetox-silencing on food choice and energy intake. **(A)** Average daily energy intake from chow, lard, 9% sucrose solution, lard and 9% sucrose solution together, total energy intake (kcal) and water intake (in ml) calculated over 1 week. **(B)** Evolution of total energy intake of both groups on HFHS free choice diet over time. Data shown as mean ± SEM. ^∗^*p* < 0.05, ^∗∗^*p* ≤ 0.01, ^∗∗∗^*p* ≤ 0.001.

Total caloric intake of the GHSR-silenced group was significantly decreased compared to the control group (9.2 ± 0.6 kcal compared to 14.0 ± 0.8 kcal; *F*(1, 13) = 22.0, *p* < 0.001) (Figure 2A). Total energy intake over time was significantly decreased in the GHSR-silenced group relative to controls (overall group effect on total energy intake: *F*(1, 11) = 15.0, *p* = 0.003) and the difference was significant at each time point from day 5 of the HFHS free choice diet until the last day of the study (Figure 2B). The caloric efficiency, however, was similar between the two groups on Day 23 of access to HFHS free choice diet (GHSR-silenced: 0.0 ± 1.1 g/kcal/day; control: −0.9 ± 2.2 g/kcal/day) (Figure 1C).

Any trends toward a lowering in body weight data in the cohort fed regular chow are unlikely to be due to an increase in energy expenditure, since we did not detect any difference in caloric efficiency (Supplementary Figure 1A).

### Silencing GHSR^*lPBN*^ Cells Alters Food Choice

Of the available foods in the free choice diet, the intake of sucrose solution was significantly lower in GHSR-silenced mice (1.2 ± 0.5 kcal) compared to controls (2.7 ± 0.5 kcal) [*F*(1, 13) = 4.74, *p* = 0.049] while that of chow and water did not differ between groups (Figure 2A). Lard intake was reduced in GHSR-silenced mice (4.7 ± 1.4 kcal) compared to controls (7.3 ± 1.0 kcal), although not reaching significance, potentially due to high variability between animals [*F*(1, 13) = 2.16, *p* = 0.166] (Figure 2A). In line with this trend, when adding data from sucrose solution and lard together (which enhances statistical power), the difference in kcal intake between the two groups was more significant than for sucrose solution alone [*F*(1, 13) = 8.99, *p* = 0.010] (Figure 2A).

GHSR-silenced and control mice had similar saccharin preference during the habituation day (Day 1) (GHSR-silenced: 61.1 ± 7.4%; control: 63.0 ± 9.3%) and the two test days (Day 2 GHSR-silenced: 86.5 ± 1.8%; control: 91.0 ± 1.7% and Day 3 GHSR-silenced: 88.2 ± 1.9%; control: 90.6 ± 2.4%).

### Co-expression of GHSR With Glutamate, PACAP, Substance P and Neurotensin in the lPBN, Revealed by RNAScope

**FIGURE 3:**
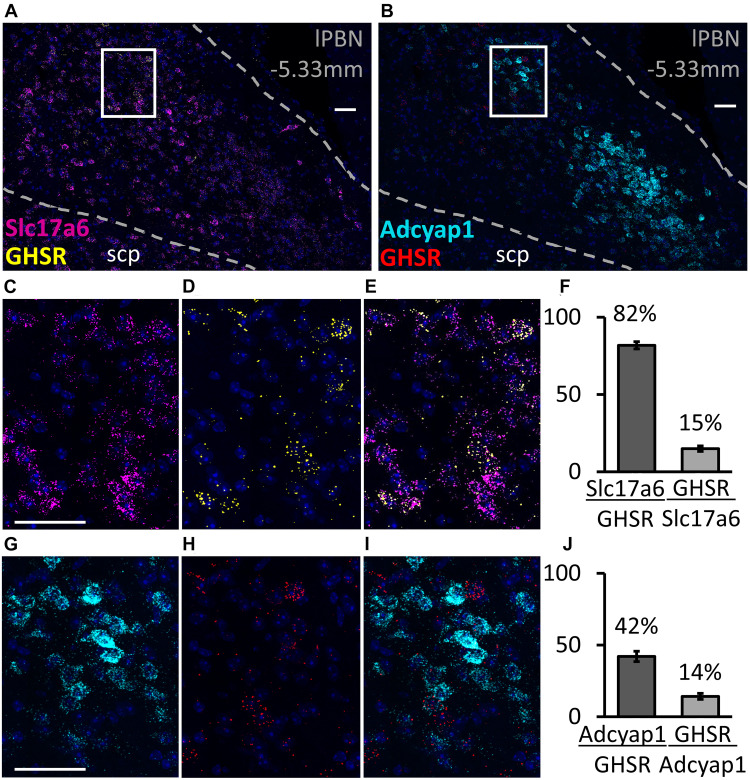
Co-expression of glutamate and pituitary adenylate cyclase-activating peptide (PACAP) with GHSR in the lPBN of mice. **(A,B)** Representative images of the lPBN with RNAscope for glutamate transporter (*Slc17a6*) and *Ghsr* mRNAs (with DAPI) and for the PACAP-coding gene (*Adcyap1*) and *Ghsr* mRNAs (with DAPI), respectively. **(C–E)** Magnifications of the indicated part of A with the signals from *Slc17a6* and *Ghsr* mRNAs separated and merged. **(G–I)** Magnifications of the indicated part of B with the signals from *Adcyap1* and *Ghsr* mRNAs separated and merged. **(F,J)** Quantification of co-expression of *Ghsr* mRNA with *Slc17a6* mRNA **(F)** and with *Adcyap1* mRNA **(J)** in the lPBN of 4 mice (5 lPBN sections per mouse). $\frac{\mathrm{Molecule}\,X}{\mathrm{Molecule}\,Y}$ indicates the percentage of MoleculeY-expressing cells co-expressing MoleculeX. Scale bar = 50 μm. scp: superior cerebellar peduncle.

**FIGURE 4:**
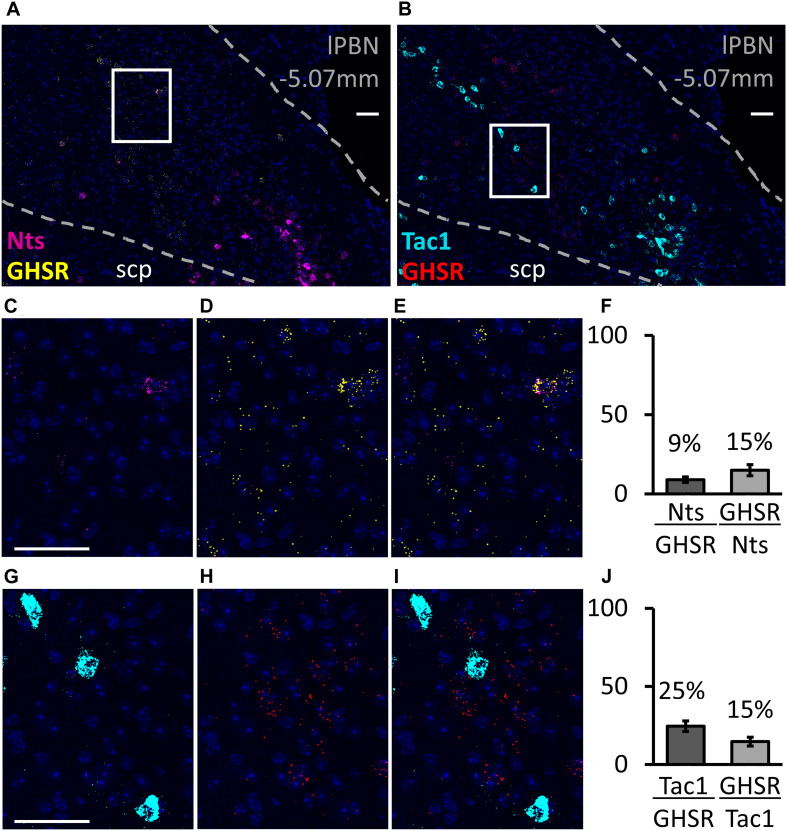
Co-expression of neurotensin and substance P with GHSR in the lPBN of mice. **(A,B)** Representative images of the lPBN with RNAscope for *neurotensin* (Nts) and *Ghsr* mRNAs (with DAPI) and for the substance P-coding gene (*Tac1*) and *Ghsr* mRNAs (with DAPI), respectively. **(C–E)** Magnifications of the indicated part of A with the signals from *neurotensin* and *Ghsr* mRNAs separated and merged. **(G–I)** Magnifications of the indicated part of B with the signals from *Tac1* and *Ghsr* mRNAs separated and merged. **(F,J)** Quantification of co-expression of *Ghsr* mRNA with *neurotensin* mRNA (*Nts*, **F**) and with *Tac1* mRNA (**J**) in the lPBN of 4 mice (4–5 lPBN sections per mouse). $\frac{\mathrm{Molecule}\,X}{\mathrm{Molecule}\,Y}$ indicates the percentage of MoleculeY-expressing cells co-expressing MoleculeX. Scale bar = 50 μm. scp: superior cerebellar peduncle.

The RNAscope study shows that, in the lPBN of mice, the majority of the GHSR-expressing cells (82.2 ± 2.1%, Figure 3) are glutamatergic (i.e., express Slc17a6 mRNA, a glutamate transporter). As many as 42.3 ± 4.2% of GHSR-expressing cells co-express Adcyap1 mRNA (i.e., PACAP; Figure 3) while 25.1 ± 3.4% co-express Tac1 mRNA (i.e., substance P; Figure 4) and 9.2 ± 2.3% neurotensin mRNA (Figure 4). Interestingly, the proportions of glutamate-, PACAP-, substance P- and neurotensin-positive cells that also express GHSR are very similar (15.1 ± 1.4, 14.4 ± 1.2, 15.2 ± 3.1 and 15.3 ± 4.1%, respectively, Figures 3, 4).

### Co-expression of GHSR and CGRP in the lPBN, Revealed by Immunohistochemistry

**FIGURE 5:**
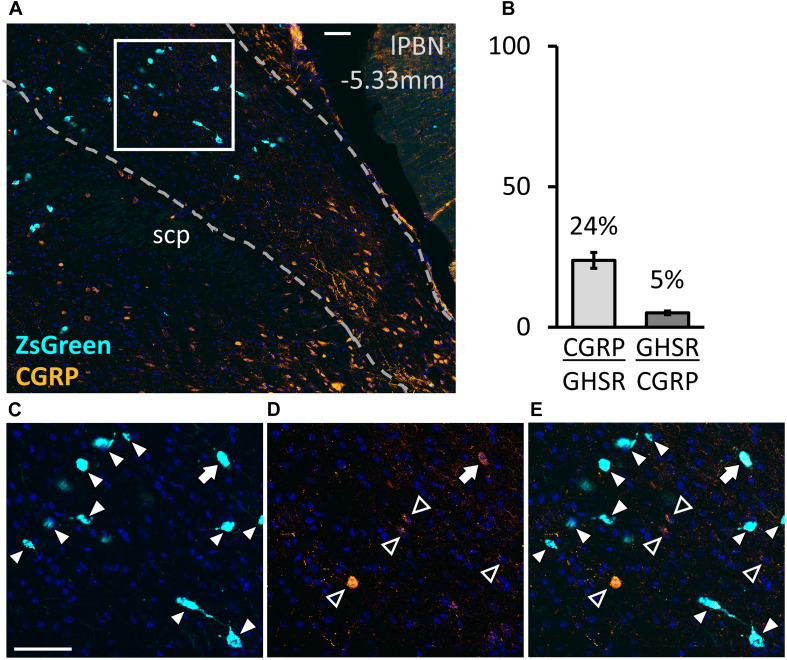
Co-expression of calcitonin gene-related peptide (CGRP) with GHSR in the lPBN of mice. **(A)** Representative image of the lPBN with immunohistochemical staining for CGRP and endogenous expression of ZsGreen in GHSR-expressing cells of *Ghsr-IRES-Cre* heterozygous mice on a ZsGreen background (with DAPI). **(B)** Quantification of co-expression of GHSR (visualized as ZsGreen) and CGRP in the lPBN of 5 mice (2 lPBN sections per mouse). $\frac{\mathrm{Molecule}\,X}{\mathrm{Molecule}\,Y}$ indicates the percentage of MoleculeY-expressing cells co-expressing MoleculeX. **(C–E)** Magnifications of the indicated part of A with the signals from CGRP and ZsGreen separated and merged. Filled arrow heads: ZsGreen-expressing cells, hollow arrow heads: CGRP-positive cells, filled arrow: cell expressing both ZsGreen and CGRP. Scale bar = 50 μm. scp: superior cerebellar peduncle.

The image analysis revealed that, in the lPBN of mice, 24.1 ± 3.2% of the GHSR-positive cells co-express CGRP and that only 5.2 ± 0.7% of CGRP-positive neurons co-express GHSR (Figure 5). Thus, the GHSR- and CGRP-expressing cells of the lPBN appear to be largely two distinct populations of cells with less than a quarter of the GHSR-positive cells also expressing CGRP.

## Discussion

Recent studies using *Ghsr-IRES-Cre* mice (Mani et al., 2017) identify the lPBN as an area with high GHSR expression in mice. Despite this, almost nothing is known about this prominent population of cells, including whether they contribute to energy homeostasis and, indeed, what their neurochemical identity might be. In the present study, using a viral approach that stops cells from signaling, we found that functional silencing of GHSR^*lPBN*^ cells protected mice from body weight gain and fat accumulation during exposure to an obesogenic HFHS choice diet. The lack of weight gain could be explained by a reduced energy intake compared to wild-type controls and did not appear to involve an increase in energy expenditure since caloric efficiency was unaltered. Colocalization studies (using immunohistochemistry and RNAscope) revealed that the majority of GHSR^*lPBN*^ cells are glutamatergic (over 80%), just over 40% express PACAP, a quarter express substance P, just under a quarter express CGRP, while just under 10% express neurotensin.

Previous studies found global Ghsr-null mice to be resistant to diet-induced obesity when introduced to a high fat diet from an early age (Zigman et al., 2005), an effect that was later reproduced by neuronal ablation of GHSR (Lee et al., 2016) and partly by ablation of GHSR specifically in the ARC AgRP neurons (by crossing AgRP-Cre and Ghsr^*f/f*^ mice) (Wu et al., 2017). Given the well-documented role of AgRP neurons in promoting food intake (Krashes et al., 2011; Essner et al., 2017), the expectation would be that deletion of GHSR in the AgRP neurons would reduce body weight and adiposity by decreasing food intake. This turned out not to be the case, however, since food intake was unaltered in these mice and their body weight phenotype was explained by an increase in energy expenditure relative to Ghsr^*f/f*^ controls, an effect believed to be mediated by an increase in non-shivering thermogenesis (Wu et al., 2017). By contrast, in the present study, the lower body weight and body fat (relative to controls) induced by silencing GHSR^*lPBN*^ cells is clearly caused by a reduced food intake. Indeed, although we did not measure energy expenditure directly, we found that caloric efficiency was unaltered. Collectively these data suggest that GHSR^*lPBN*^ cells might be more relevant for diet-induced hyperphagia than for energy expenditure.

Since our mice were fed a HFHS free choice diet, it was possible to explore their dietary preference. We found that mice with silenced GHSR^*lPBN*^ cells consumed significantly less sucrose solution relative to control mice. We did not detect any significant reduction in lard intake, likely due to high variability between the mice and also the small number of animals studied. However, when we explored caloric intake of sucrose plus lard together, the difference of intake of these combined palatable foods reached a higher significance level than for sucrose alone, comparing mice with silenced GHSR^*lPBN*^ cells and controls. Chow intake was not altered by silencing of GHSR^*lPBN*^ cells, when offered in the choice diet or as a single food (Supplementary Material 1). Thus, GHSR^*lPBN*^ silencing appears to suppress palatable food intake and may not be selective for sucrose or lard.

It would appear that the impact of silencing the GHSR^*lPBN*^ cells on food choice in mice (i.e., decreased palatable food intake) is not diametrically opposite to that of central ghrelin injection in rats (i.e., increased intake, especially of chow) (Schéle et al., 2016; Bake et al., 2020). This is perhaps not surprising since these GHSR^*lPBN*^ cells are unlikely to be the only cells engaged by ghrelin to alter food choice and may include, for example, direct effects of ghrelin at the level of the VTA (Schéle et al., 2016). Ghrelin injection and GHSR^*lPBN*^ silencing are also very different techniques and there may be different levels of recruitment of neighboring GHSR populations controlled in Tetox-silencing (this study) versus a ghrelin injection (Schéle et al., 2016; Bake et al., 2020). Tentatively, these collective data would lend support to the hypothesis that in situations of energy defect (when ghrelin receptor signaling is high), ghrelin recruits pathways (including lPBN) to promote intake of regular chow but when fed (and ghrelin receptor signaling is low), it promotes intake of palatable foods that escape energy needs.

Although the effects on food choice appeared to be linked to decreased intake of palatable food, we could not detect a change in preference for a non-caloric sweet taste (saccharin), tested using a single saccharin concentration (Rabasa et al., 2016). Further tests exploring, for example, threshold saccharin preference and the expression of sweet taste receptors, would be required in order to make firm conclusions regarding this result. Tentatively, these data could suggest that the decrease of sucrose intake induced by silencing GHSR^*lPBN*^ cells is not due to an impairment of sweet taste sensation. The role of the PBN as a relay for taste/gustatory information was established many decades ago (Norgren and Leonard, 1971, 1973; Norgren and Pfaffmann, 1975). Yet, the lPBN is also involved in the hedonic valuation of food (Hajnal and Norgren, 2005; Scott and Small, 2009) and in the regulation of palatable food intake (De Oliveira et al., 2011; Rodriguez et al., 2019). The GHSR^*lPBN*^ cells studied here, therefore, are likely part of the neurocircuit regulating consumption of palatable food and food choice but we did not yet find evidence for involvement in the sensing or relaying of information related to sweet taste.

The technique used here to silence lPBN cells involves viral vector-mediated delivery of tetanus toxin light chain specifically to GHSR-expressing cells in this area. Neighboring areas are unlikely to have been targeted by this viral vector, in part due to the rather isolated location of the lPBN, but also because GHSR is not expressed in neighboring areas with the possible exception of the medial PBN (mPBN) (Mani et al., 2017), that is implicated in taste-related behaviors (Chiang et al., 2019). The fact that silencing the GHSR^*lPBN*^ cells in this way caused a phenotype identifies these cells as having a role in this phenotype. Arguably, we do not know if GHSR is the key signal in these cells for the effects observed, since the cells will no longer respond to any afferent signal. Activation by PYY of the Y1 receptor expressed in the lPBN was shown to also increase food intake, for example (Alhadeff et al., 2015). Yet, evidence that GHSR may be the critical signal here is supported by our previous data demonstrating an orexigenic role for ghrelin at the level of the lPBN; we found that ghrelin delivery to the lPBN of rats caused an increase in food intake and an altered food choice (Bake et al., 2020). Additionally, a very recent study showed that the orexigenic effects of intra-PBN ghrelin does not occur in GHSR KO mice (Zhang et al., 2020). Other potential candidate neuronal populations, shown to induce a feeding response upon activation in this area, include those expressing benzodiazepine receptors (Higgs and Cooper, 1996; Söderpalm and Berridge, 2000), μ-opioid receptors (Wilson et al., 2003) and cannabinoid 1 receptors (Dipatrizio and Simansky, 2008). It would be of interest to determine whether the GHSR^*lPBN*^ cells co-express these receptors or indeed receptors for other appetite-regulating hormones, such as GLP-1, PYY and leptin (Merchenthaler et al., 1999; Scott et al., 2009; Alhadeff et al., 2015). Of these, it could be especially interesting to explore co-expression between GLP-1 receptor and GHSR, since there is some overlap in distribution, at least in the dorsal part of the lPBN (Merchenthaler et al., 1999; Zigman et al., 2006).

We further demonstrate here using the RNAscope technique (to simultaneously probe multiple mRNAs) that the GHSR^*lPBN*^ cells are a heterogeneous population within which the majority of the cells are glutamatergic, slightly less than half co-express PACAP and small proportions are substance P- and neurotensin-positive. Moreover, a surprising and interesting fact is that, GHSR is expressed on approximately 15% of the cells from each neuropeptide/neurotransmitter-expressing population studied (namely expressing glutamate, PACAP, substance P or neurotensin). This might point toward an important role of GHSR in modulating different circuits in the lPBN. Regarding the potential circuitry of the GHSR^*lPBN*^ cells, Niu and colleagues identified glutamatergic lPBN neurons that project to orexin-expressing hypothalamic neurons in rats (Niu et al., 2010) that are orexigenic (Sakurai et al., 1998). In addition, PACAP neurons in the lPBN, which are also glutamatergic, were shown to project to the bed nucleus of the stria terminalis (BNST) and the CeA and to be of importance in the mediation of pain (Missig et al., 2014, 2017). The ghrelin system, on the other hand, has been suggested to have pain-reducing functions, inhibiting inflammatory and neuropathic pain (Sibilia et al., 2006; Vergnano et al., 2008; Kyoraku et al., 2009; Guneli et al., 2010). The presence of GHSR expression in PACAP^*lPBN*^ cells might, thus, point toward the fact that GHSR signaling in these cells modulate pain transmission.

Finally, given the fact that CGRP neurons in the lPBN have such a prominent role in feeding control (Carter et al., 2013; Campos et al., 2016), we sought to determine, by immunohistochemistry, whether there is colocalization between CGRP and GHSR in the lPBN of *Ghsr-IRES-Cre* mice. We found that the GHSR- and CGRP-expressing cells of the lPBN are mostly separate populations, but that a quarter of the GHSR-expressing cells also express CGRP. Thus, the orexigenic GHSR^*lPBN*^ cells and the well-known anorexigenic circuit in the lPBN are mainly distinct, but a few cells seem to be common to both. Interestingly, CGRP has been shown to be expressed on at least some of the PACAP^*lPBN*^ neurons projecting to the CeA and BNST (Missig et al., 2014), and similarly to CGRP signaling in the CeA, PACAP signaling in the BNST was found to produce anorexia and body weight loss in rats (Kocho-Schellenberg et al., 2014). It is possible, therefore, that GHSR is expressed by lPBN cells that are both CGRP- and PACAP-positive and that GHSR signaling serves to modulate the transmission of these anorexigenic signals in the lPBN.

In summary, our study shows that GHSR^*lPBN*^ cells have a role in HFHS diet-induced hyperphagia and body weight gain and also influence food choice. Indeed, given that there is much redundancy and overlap in the neurocircuitry controlling feeding behaviors, the fact that we observed these effects merely by silencing this GHSR^*lPBN*^ cell population, suggests they may have a rather important role in feeding behavior and energy balance control. Our work also provides the first evidence that the GHSR^*lPBN*^ cells form a heterogeneous cell population with a great proportion of them co-expressing glutamate and PACAP, and only a small proportion containing substance P, neurotensin and CGRP - the CGRP cells being a well-studied anorexigenic population in this region (Carter et al., 2013; Campos et al., 2016). Taken together with other recent publications (Bake et al., 2020; Zhang et al., 2020), it now seems clear that the brain pathways engaged by ghrelin for its effects on feeding control and energy balance include the lPBN, and it will be important to explore the contribution of the lPBN relative to other key targets that express GHSR, such as the arcuate nucleus and the ventral tegmental area, (Skibicka and Dickson, 2011) for these effects.

## Data Availability Statement

The raw data supporting the conclusions of this article will be made available by the authors, without undue reservation.

## Ethics Statement

The animal study was reviewed and approved by Ethics Committee for Animal Care and Use at the University of Gothenburg (permit number: 27-2015 and 132-2016).

## Author Contributions

The work was undertaken in the group of SD, who is the lead scientist of this study. ML performed the experiments and analyzed the data with assistance from FP-S, IS, ES, and TB. ML, FP-S, RA, and SD designed the studies. ML wrote the manuscript with guidance from SD and FP-S. All authors contributed to the article and approved the submitted version.

## Conflict of Interest

The authors declare that the research was conducted in the absence of any commercial or financial relationships that could be construed as a potential conflict of interest.

## Footnotes

1 http://mouse.brain-map.org

2 http://mouse.brain-map.org
