# Supplementary material for: Functional and Neurochemical Identification of Ghrelin Receptor (GHSR)-Expressing Cells of the Lateral Parabrachial Nucleus in Mice
Source: Front Neurosci. 2021 Feb 15;15:633018. doi: 10.3389/fnins.2021.633018 (PMC7917048; doi:10.3389/fnins.2021.633018)
Supplement: Supplementary file 1 [file Table_1.docx]

**Supplementary Material**

**Supplement 1: Silencing GHSR^lPBN^ cells does not alter food intake or body weight in mice fed a standard chow diet**

Here, we sought to determine the effect of silencing the GHSR^lPBN^ cells on the evolution of food intake and body weight when mice are fed standard chow under the same period as for the mice fed the high-fat high-sugar diet (i.e. starting 1 week after surgery and for 23 days). Note that the timeline differs slightly from the mice fed a HFHS diet, since these animals continuously received chow, whereas the HFHS diet commenced one week after surgery.

**Materials and Methods**

Eleven heterozygous Ghsr-IRES-Cre (Ghsr-Cre Het) male mice and 9 wild-type male littermates (WT) were injected bilaterally into the lPBN with a viral vector (AAV1-CBA-DIO-eGFP-Tetox-WPRE-pA) (Carter et al., 2015) following an identical protocol to that stated in the “stereotaxic surgeries” section of the material and methods in the main text. The GHSR^lPBN^ cells were, therefore, silenced in the Ghsr-Cre Het mice (now referred to as the GHSR-silenced group) but not in the WT controls. To investigate the effect of silencing GHSR^lPBN^ cells on food intake and body weight when mice are given a standard chow diet, chow intake and body weight were measured 2-3 times a week, at the same time of the day (approximately at 12:00) for 23 days after surgery. In addition, caloric efficiency was calculated on Day 23 as follows: caloric efficiency = (body weight gain (g) / food intake (kcal)) x 100 (Rabasa et al., 2019). Five of the GHSR-silenced mice were excluded because post-mortem evaluation revealed that the injection site was off-target. One additional GHSR-silenced mouse was identified as an outlier by the SPSS software in the caloric efficiency data. Thus, the final analysis of body weight and food intake included 6 GHSR-silenced mice and 9 control mice and the analysis of caloric efficiency included 5 GHSR-silenced mice and 9 controls.

Data were analyzed using IBM SPSS Statistics 25 (IBM Corp., Armonk, NY, USA). All data were tested for normal distribution using a Shapiro-Wilk test and for homogeneity of variances using a Levene’s test. Throughout the 23 days of chow diet exposure, cumulative kcal intake and % body weight gain were analyzed by one-way repeated measures ANOVA with the measurement days as the within-subject factor. Caloric efficiency was analyzed using an independent samples Student’s t-test.

**Results**

The cumulative energy intake (kcal) from chow did not differ between GHSR-silenced and control mice (overall group effect on cumulative kcal intake: *F* (1, 13) = 0.00, *p* = 0.995) (Supp. Fig. 1A). Similarly, there was no difference in percentage body weight gain between the two groups over time (overall group effect on percentage body weight gain: *F* (1, 13) = 1.94, *p* = 0.187) (Supp. Fig. 1B). Finally, caloric efficiency was not affected in the GHSR-silenced mice (0.4 ± 0.5 g/kcal/day) compared to the controls (1.2 ± 0.8 g/kcal/day) (*t*(12) = 0.75, *p* = 0.470, Supp. Fig. 1A).


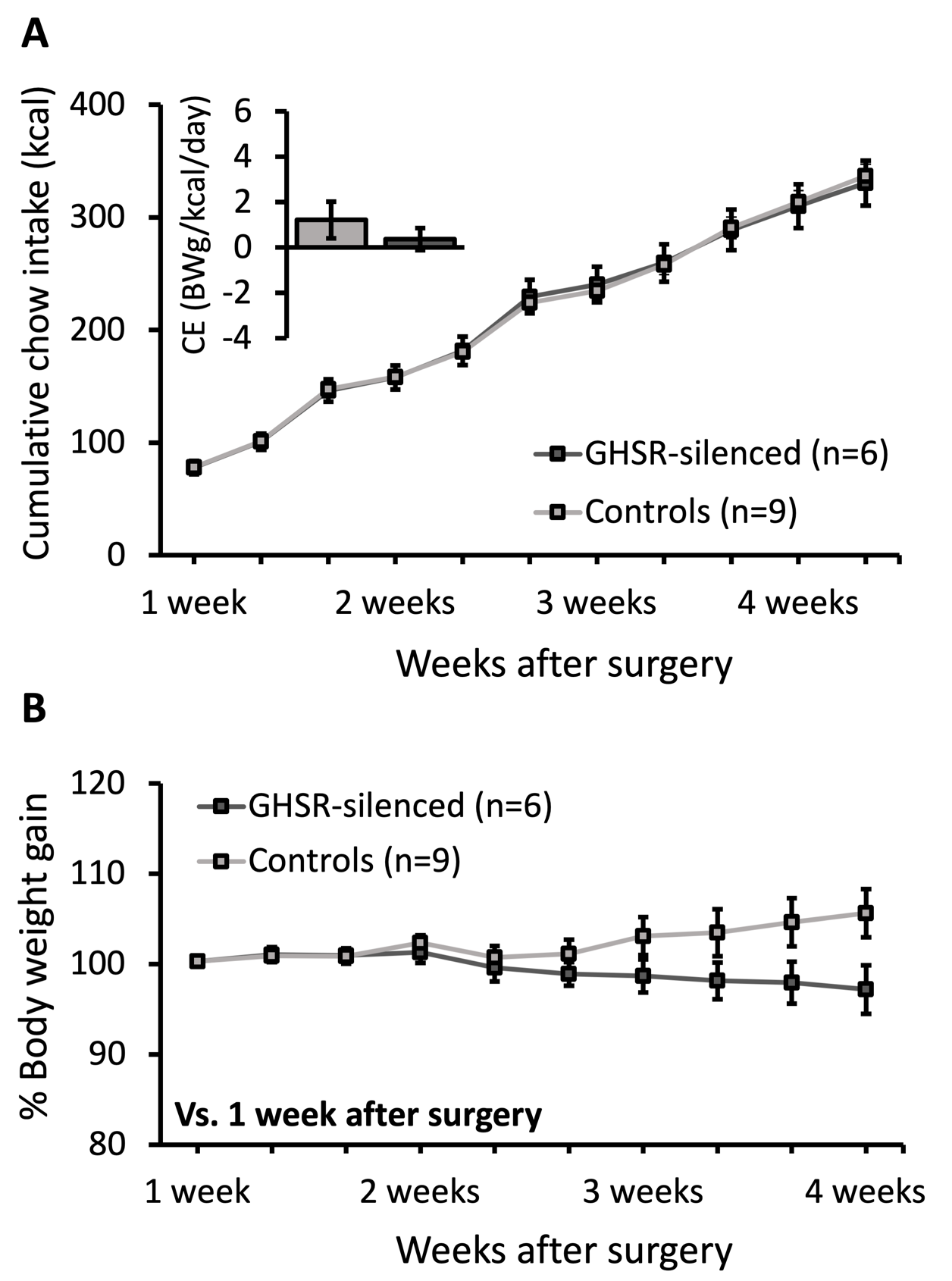


**Supplementary Figure 1.** **Effect of GHSR^lPBN^ cells Tetox-silencing on chow intake and body weight. (A)** Cumulative chow intake in kcal over time and caloric efficiency (CE) on day 23 after surgery for the GHSR-silenced and the control groups **(B)** Evolution of % body weight gain of both groups on standard chow diet over time (body weight at 1 week after surgery = 100 %). Data shown as mean ± SEM.

**References**

Carter, M.E., Han, S., and Palmiter, R.D. (2015). Parabrachial calcitonin gene-related peptide neurons mediate conditioned taste aversion. J Neurosci 35**,** 4582-6.

Rabasa, C., Askevik, K., Schele, E., Hu, M., Vogel, H., and Dickson, S.L. (2019). Divergent Metabolic Effects of Acute Versus Chronic Repeated Forced Swim Stress in the Rat. Obesity (Silver Spring) 27**,** 427-433.
